# A triple-punch approach: methionine restriction enhances combination inhibitors in brain metastatic triple-negative breast cancer

**DOI:** 10.1172/JCI193171

**Published:** 2025-07-01

**Authors:** Samyuktha Suresh, James M. Ford

**Affiliations:** Department of Medicine, Division of Oncology, Stanford University, Stanford, California, USA.

## Abstract

Triple-negative breast cancer (TNBC), the most aggressive subtype of breast cancer, presents a clinical challenge in developing effective treatment options. In this issue of the *JCI*, Zeng et al. demonstrate a provocative and promising therapeutic strategy for TNBC by leveraging the metabolic vulnerabilities presented by methylthioadenosine phosphorylase (MTAP) deletion to genotoxic stress inducers, such as poly (ADP-ribose) polymerase inhibitors (PARPi). They found that combining *MTAP* deletion or inhibition with PARPi was highly effective in brain metastatic TNBC where the methionine-limited environment further enhanced this combination. This approach underscores the importance of targeting metabolic vulnerabilities in the development of personalized cancer therapies.

## Challenges in triple-negative breast cancer treatment

Triple-negative breast cancer (TNBC) is a particularly aggressive subtype of breast cancer, accounting for approximately 15% of all breast cancers but nearly 25% of breast cancer–related deaths (1). The aggressive nature of TNBC is attributed to its high rates of metastasis, particularly to the brain and other distant organs, and its tendency to relapse after initial treatment (2). Another important characteristic of TNBC is its intratumor and intertumor heterogeneity, further complicating the development of universal treatment approaches (1). Therefore, effective treatment strategies for patients with TNBC remain an unmet clinical need.

## Exploiting genetic vulnerabilities in TNBC

One of the most promising avenues of research in TNBC treatment involves targeting specific genetic and metabolic vulnerabilities within TNBC cells. Recent studies have focused on the role of methylthioadenosine phosphorylase (MTAP), an enzyme involved in the methionine salvage pathway. *MTAP* is frequently deleted in various cancers, including TNBC, often as a codeletion event with cyclin-dependent kinase inhibitor 2A (CDKN2A) (3). *MTAP* deletion occurs in approximately 15% of all cancers and is particularly prevalent in certain solid tumors, such as gliomas, non–small cell lung cancer, and pancreatic cancer (3). Although *MTAP* deletion occurs in less than 5% of breast cancers overall, its loss may be closer to 10% in TNBC (4).

In this issue of the *JCI*, Zeng et al. (5) demonstrate that *MTAP* deletion sensitizes TNBC cells to DNA damaging genotoxic agents, particularly poly (ADP-ribose) polymerase inhibitors (PARPi). PARPi have emerged as a promising synthetic lethal strategy for cancers with mutations in the DNA damage repair genes, *BRCA1* and *BRCA2* (6, 7), and perhaps others involved in homologous recombination, such as *PALB2* (8). However, the clinical application of PARPi in TNBC has been limited due to the low frequency of these mutations. The findings of this study suggest that *MTAP* deletion may serve as a potential biomarker for identifying additional patients with TNBC who could benefit from PARPi treatment (5).

**Figure 1:**
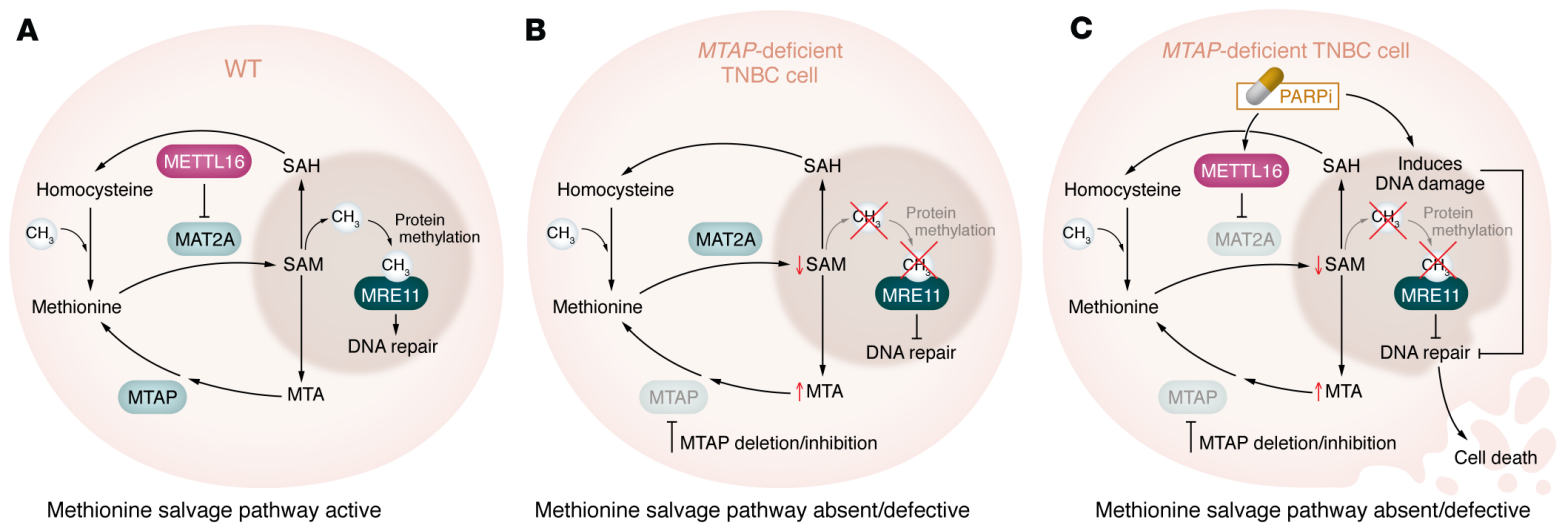
TNBC cells with *MTAP* deficiency possess impaired methionine metabolism that disrupts DNA repair. (**A**) In WT cells, the methyl donor SAM participates in various biological processes, including DNA repair. (**B**) TNBC cells lacking MTAP show disrupted methionine metabolism, which depletes SAM and impairs the recruitment of MRE11. Without MRE11, and in the context of a methionine-deplete environment such as in the brain, DSBs remains unrepaired. (**C**) In *MTAP*-deficient TNBC cells, PARPi activates METTL16, which further restricts SAM activity and compounds the effect of SAM on decreased DNA repair. SAH, S-adenosylhomocysteine; MTA, methylthioadenosine.

Mechanistically, Zeng et al. (5) showed that *MTAP* deficiency disrupted methionine metabolism, depleting s-adenosylmethionine (SAM) levels. SAM is a critical methyl donor involved in various biological processes, including DNA repair (Figure 1A). The depletion of SAM impaired the recruitment of MRE11, a DNA end resection protein, to sites of DNA damage (9) (Figure 1B). This impairment compromised the cell’s ability to repair DNA double-strand breaks (DSBs), rendering *MTAP*-deficient TNBC cells more susceptible to the cytotoxic effects of PARPi (Figure 1, B and C). This study also highlights a feed-forward loop between methionine metabolism and DNA repair mechanisms (5). It has previously been shown that SAM levels regulate the expression of MAT2A, the enzyme that converts methionine to SAM (10). Low SAM levels typically trigger an upregulation of MAT2A by inducing its splicing via METTL16 (10). As *MTAP* deletion leads to reduced SAM levels, the increase in MAT2A is suppressed by PARPi, which activates METTL16 (Figure 1C), further exacerbating the effect on DNA repair (5). This “one-two punch” mode of treatment is emphasized by in vitro and in vivo experiments demonstrating strong synergy between *MTAP* deletion or inhibition and PARP inhibition. A key finding of this study is that when methionine was restricted in a setting with *MTAP* deletion or inhibition combined with PARP inhibition (5), this triple-punch approach enhanced MTAP inhibitor–PARPi, synergy, leading to cell death and a decrease in tumor volume. This finding is particularly relevant in the context of brain metastatic TNBC, where the inherent methionine-limited environment further enhances the efficacy of combining MTAP deletion or inhibition with PARPi (5).

Zeng and colleagues demonstrate that, by leveraging the interplay between methionine metabolism and DNA repair, a promising personalized treatment strategy can be developed for patients with TNBC — one that might provide a so-to-speak “knockout” blow to TNBC brain metastases, in particular.

## Conclusions and future perspectives

The report by Zeng et al. (5) highlights the critical role of *MTAP* deletion in enhancing TNBC’s sensitivity to PARPi, providing a compelling rationale for exploring metabolic vulnerabilities as therapeutic targets. By integrating insights from cancer metabolism with established treatment strategies, this study paves the way for developing innovative approaches to treating TNBC. Moreover, the implications extend beyond TNBC, as the insights gained from understanding the role of *MTAP* in cancer metabolism may inform therapeutic strategies for other malignancies characterized by similar metabolic alterations.

Approaches to synthetic lethality pose a potentially powerful way to discover targeted therapies based on our expanding understanding of cancer mutations and genomics (11). While leading to the initial development of PARPi for *BRCA1/2* mutant tumors, robust methods for systematically identifying synthetic lethal genes are lacking. However, recent unbiased screens can facilitate the identification of gene pairs involved with synthetic lethality in human cancer cells. In addition, the rational design of synthetic lethality discovery, as demonstrated here, may lead to novel combinations of agents and physiologic conditions relevant for specific cancer subtypes and situations. Furthermore, the preclinical discovery of such synergistic combinations is critical for feeding the pipeline for emerging early clinical trials of effective combination therapies in genomically characterized human tumors (12).

This research also raises important questions regarding the broader implications of targeting metabolic vulnerabilities in cancer therapy. As our understanding of cancer metabolism continues to evolve, it is crucial to consider how metabolic alterations can influence treatment responses and resistance mechanisms beyond traditional genetic targets. Future studies should aim to elucidate the complex interactions between metabolic pathways and established therapeutic targets and to develop combination therapies that can effectively overcome resistance and improve patient outcomes.

## Version 1 07/01/2025

Electronic publication
